# Assessing immunotherapy response: going beyond RECIST by integrating early tumor growth kinetics

**DOI:** 10.3389/fimmu.2024.1470555

**Published:** 2024-12-20

**Authors:** Mehdi Felfli, Alexandre Thinnes, Sebastien Jacques, Yan Liu, Antoine Iannessi

**Affiliations:** ^1^ Median Technologies, Imaging Lab, Valbonne, France; ^2^ Centre Antoine Lacassagne, Radiology Department, Nice, France

**Keywords:** growth rate, non-small cell lung cancer, immunotherapy, RECIST 1.1, novel Gompertz model, progression-free survival, time to response, treatment response patterns

## Abstract

**Objective:**

Assess the contribution of early tumor growth dynamics modeling to predict clinical outcomes in non-small cell lung cancer patients receiving immunotherapy, alongside standard RECIST 1.1 criteria.

**Methods:**

Our retrospective studies used data from 861 patients with advanced NSCLC enrolled in three randomized Phase III trials evaluating immunotherapy plus chemotherapy were analyzed. Tumor size measurements up to two follow-up time points were used to fit a novel Gompertz model and estimate growth rate (GR) and kinetic parameters representing depth of response (A), speed of response (B), and long-term modulation (M). Correlations between these early tumor growth parameters and clinical outcomes such as progression-free survival (PFS) and time to response (TTR) were assessed. Descriptive and discriminative analyses were performed to delineate tumor growth dynamics across various response profiles based on RECIST 1.1 criteria.

**Results:**

The novel Gompertz model accurately described early tumor growth kinetics in 861 non-small cell lung cancer patients treated with immunotherapy. Lower growth rate (GR) and model parameter M were associated with longer progression-free survival (PFS) (HR=0.897 and 7.47x10^-7, respectively). Higher GR and parameter A correlated with shorter time to response (HR=0.575 and 0.696, respectively). Responders had significantly lower A (p=1.51e-53) and higher GR (p=0.4e-12) than non-responders. Non-durable stable disease patients had higher GR (p=0.0001) and parameter B (p=0.0002) compared to late responders. Early tumor growth parameters showed potential for predicting long-term outcomes and treatment response patterns.

## 1 Introduction

Immunotherapy has revolutionized cancer treatment, leading to improved survival for many patients, but a large proportion of patients don't benefit from these treatments and a large panel of potential drug need to be tested (1). Moreover, Immunotherapy has been shown to induce unique and complex patterns in the kinetics of tumor response, including pseudo progression and hyper progression, which differ from the typical responses observed with traditional oncotherapy making It more difficult to apprehend (2).

Several variations of common efficacy assessment based on images have been developed to address the clinical complexities, such as the iRECIST or iRANO criteria (3–5). These methods lack the inclusion of a direct measurement of tumor growth rate as a biomarker. In parallel, tumor growth modeling (TGM) has gained increasing attention in recent years in the characterization of drug effects on tumor size and identify prognostic and predictive factors for overall survival. Various mathematical models have been proposed to describe tumor growth, including exponential, logistic, Gompertz, and von Bertalanffy models (6, 7). These models can be used to estimate tumor growth rate (TGR), providing a quantitative assessment of change in tumor volume over time.

The mechanism of action of immunotherapy potentially limits the use of tumor growth markers for assessing treatment efficacy due to its indirect action based on patient immunity. However, the current published data suggests that this type of marker can also be used for prognostic or predictive purposes correlated with treatment response and overall survival (OS) in patients with various types of cancer and treatment strategies including immunotherapies (8–12).

In this paper, we aim to retrospectively analyze coherent data from clinical trials for the evaluation of immune therapy efficacy to estimate the validity of tumor growth models in these patients and assess the contribution of a tumor dynamics analysis compared to a standard RECIST 1.1 analysis.

### 1.1 Background

Tumor growth models are mathematical representations of tumor growth over time. These models can be broadly classified into linear or non-linear models including logistic family models. Linear models assume a constant growth rate and are often used to describe early tumor growth (13). Non-linear models, on the other hand, account for changes in growth rate over time and can capture more complex growth patterns (7). The logistic family of models, which includes Gompertz and von Bertalanffy models, assumes that tumor growth follows a sigmoidal curve, with an initial phase of exponential growth followed by a decline of growth as the tumor approaches its carrying capacity (7). Understanding the strengths and limitations of these different model types is essential for selecting the appropriate model for a given dataset and research question.

In the context of immunotherapy and oncotherapy, TGM can provide important insights into the growth dynamics driven by the intricate interplay between tumors and the immune system, thereby facilitating the early identification of response predictors or progression markers (12, 14, 15). For example, changes in tumor size at 12 weeks have been shown to predict survival outcomes in patients receiving immunotherapy (16). While traditional response criteria like RECIST play a role in evaluating treatment efficacy (17, 18), they often fail to capture the complexities of treatment response, particularly in immunotherapy (19–21). Growth kinetics modeling offers a complementary approach that can capture the longitudinal dynamical course of tumor size and provide more in-depth discrimination for response patterns.

Most of the studies considering tumor growth as biomarker compare two periods of interest i.e., pre / post treatment or two cohorts i.e., treatment arm / control arm (11, 18). However, for central analysis purposes during clinical trial it can be difficult to retrieve pre-baseline assessment as per definition those examinations are not part of the prospective evaluation. Considering this important limitation on the use of kinetic biomarkers, our approach has been more practical and considers only one period of interest i.e., the early kinetic phase after treatment initiation.

The goal of our analysis is 1) to determine whether there is practical biomarker value in including TGM in a standard RECIST 1.1 efficacy analysis; and 2) for which patients this analysis can be helpful.

## 2 Materials and methods

### 2.1 Material

#### 2.1.1 Study population

The study population included patients with non-small cell lung cancer (NSCLC) at stages IIIB and IV who were enrolled in three randomized, double-blind, multicenter, phase III clinical trials. Patients received a combination of anti-PD1/PD-L1 therapy and chemotherapy (I+Chem) and underwent RECIST 1.1 evaluations every 6-9 weeks depending on the trial protocol (see: **Table 1**).

**Table 1:** Trials included in the retrospective study were phase 3 efficacy trials for locally advanced or metastatic NSCLC (stage IIIb and IV) with progression free survival as primary endpoint and best overall response as secondary endpoint.

Trial	Treatment	Patient Number	Assessments Frequency	Mean Duration
Trial 1	Immunotherapy (anti PD1/PD-L1) + Chemotherapy vs Chemotherapy alone	645	Every 6 weeks	29.5 weeks
Trial 2		391	Every 9 weeks	35 weeks
Trial 3		139	Every 6 weeks	24 weeks

At least three timepoints (TP) including the baseline and measurable disease were considered to compute early tumor growth kinetics (TGK) this was made to accurately capture early response patterns and model non-linear growth dynamics. Patients with incomplete data or those who did not meet the above eligibility criteria were excluded from the analysis. A total of 861 patients were available for analysis. Neither overall survival data nor treatment arm indication were available at the time of the analysis.

**Figure 1:**
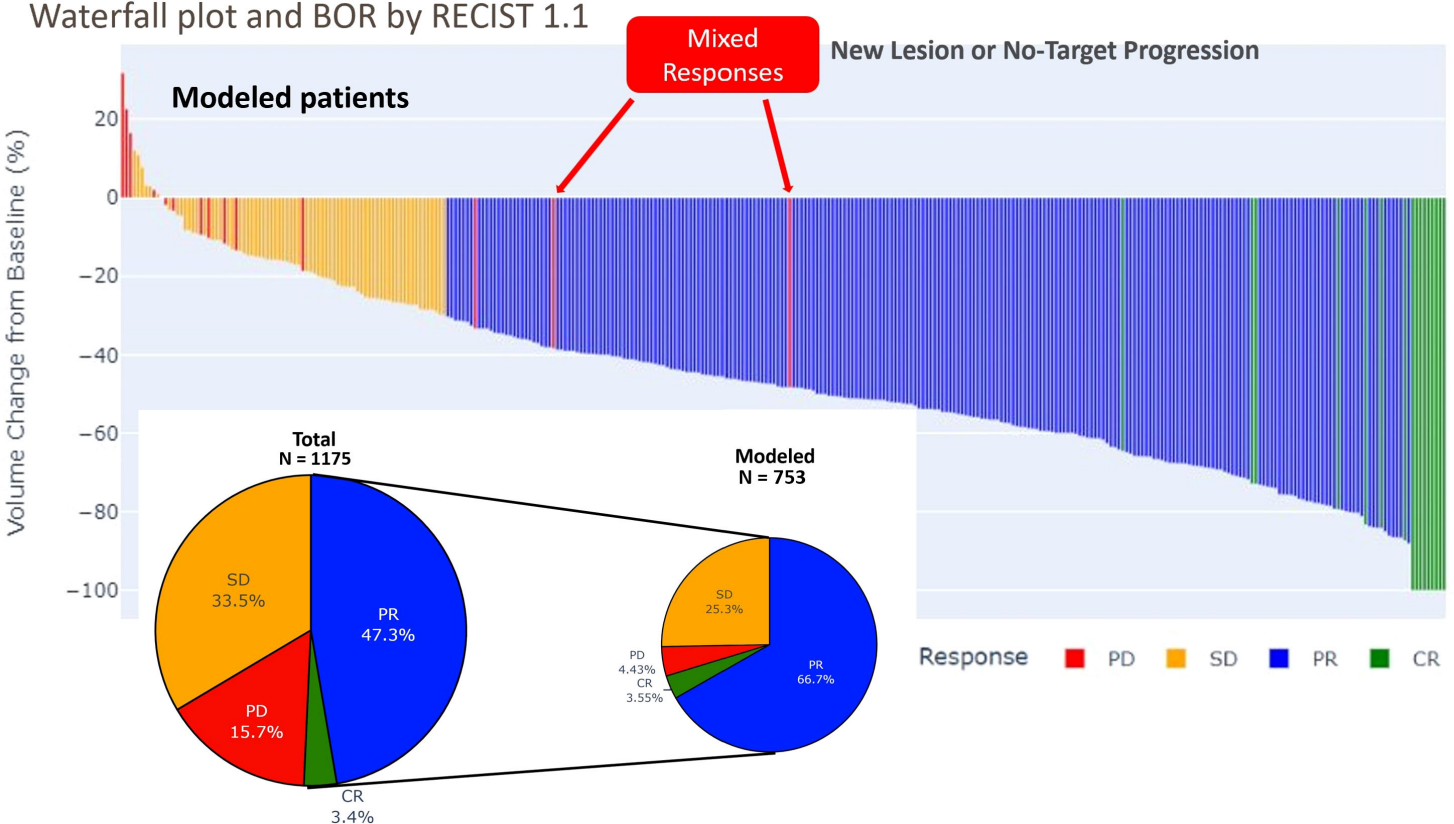
Waterfall plot and pie plot of best overall response (BOR) by RECIST 1.1 in total and modeled set of patients: The waterfall plot displays the percentage of change in total tumor size from baseline for each patient, arranged in order of decreasing response. Only a few patients experienced increase of the targets tumor burden while the 2/3 of the patients experienced a tumor shrinking leading to response. Some patients experienced a progression on non-target lesion, due to new lesion while their tumor burden has been diminishing (mixed response).

Among those eligible patients, the mean baseline sum of diameters (SOD) was 74.61 mm. Using Best overall response (BOR), The responders (CR/PR) represented 70% of the population while around 4% only of patients experienced progression during the course of the trials. BOR proportions for modeled and total patients are displayed in **Figure 1**.

**Figure 2:**
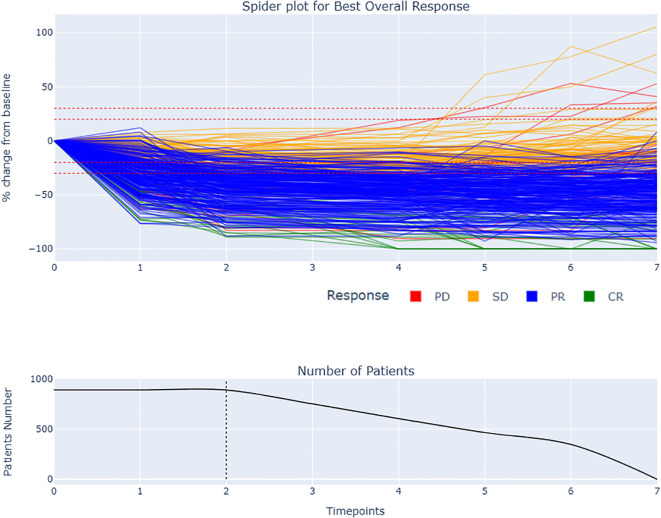
Spider plot and BOR by RECIST 1.1 with remaining number of patients during the late period of analysis: The spider plot displays the change in total tumor size over time for each patient. The responders had generally more rapid and sustained decrease in tumor size compared to stable disease. The dotted line at two follow-up time points indicates the early window period of analysis used to compute the tumor growth modeling. Some patients experienced a delayed response, with an initial increase in tumor size followed by a decrease.

The duration of response (DOR) had a mean of 26.53 weeks, with a minimum of 12 weeks and a maximum of 36 weeks. The time to response (TTR) had a mean of 15.54 weeks, with a minimum of 6 weeks and a maximum of 36 weeks. The progression free survival (PFS) had a mean of 30.29 weeks, with a minimum of 6 weeks and a maximum of 36 weeks. These results suggest that the treatment is effective in achieving a response in patients, with a relatively long duration of response and progression free survival. However, there is variability in the time to response and progression free survival, indicating that some patients may respond more quickly or have longer lasting responses than others (see **Figures 1**, **2**). These observations are consistent with previous studies using RECIST 1.1 to assess response in solid tumors (22, 23).

##### 2.1.1.1 Inclusion criteria

All participants must meet all the following criteria to be eligible for enrollment in the study.

- 18 years
- Chest CT scan for the diagnosis of NSCLC
- Pathology-confirmed NSCLC
- At least one measurable lesion (≥1 cm) per RECIST 1.1 in lung
- Chest CT scans with slice thickness ≤5mm for each FUTP

##### 2.1.1.2 Exclusion criteria

- Patients with less than 3 timepoints

### 2.2 Methods

#### 2.2.1 Period of interest and patterns of responses

For the reasons mentioned previously, we defined only one period of interest i.e., “the early phase after treatment initiation” for the kinetic analysis. We arbitrarily considered a two follow-up time points period after treatment initiation to collect enough kinetic information and used that information for TGM. Conversely, we determined a late period phase beginning after 3 follow-ups that was used to verify predictive performance of TGM parameters.

Considering those two periods, we performed a classification of patterns of response according to RECIST 1.1 as defined in **Table 2**.

**Table 2:** Classification of tumor response patterns in early and late phases of immunotherapy follow-up based on RECIST 1.1 criteria.

Early Phase After Treatment (=<12w)	Late Phase (>=18w of follow-up)
Progression per RECIST 1.1	
Hyperprogression^*^ (HpPD)	
Paradoxical Progression^**^ (PaPD) (mixed response)	
PseudoProgression^***^ (PsPD)	
Stable Disease per RECIST 1.1	Durable^x^ SD
	Non-Durable^#^ SD
	Late Response
Response (PR/CR) per RECIST 1.1	Durable^x^ PR/CR
	Non-Durable^#^ PR/CR
	Paradoxical Progression^***^

*HpPD was defined as progression with a 2x superior to median growth rate within progressor patients. ** PaPD was defined as patients who achieved CR, PR within targets lesion but are in progression because of new lesions or non-target lesion *** PsPD was defined as progression with patients who had PD at early phase but subsequently achieved CR, PR in the next two follow-up time points; x Durable was defined as patient with early phase RECIST status remaining stable along the late phase. # Non-Durable was defined as patient with event of progression during the late phase.

#### 2.2.2 Tumor growth modeling approach

Mathematical tumor growth models have been used in various imaging-based clinical trials to better understand tumor growth kinetics, assess treatment response, and predict patient outcomes.

The choice of tumor growth modeling approach is a critical step in predicting the response of cancer therapies (6, 7).

The Gompertz model describes the growth of tumors over time. It assumes that the growth rate of the tumor is initially slow, then increases exponentially before eventually decreasing and approaching zero as the tumor reaches its carrying capacity.

**Figure 3:**
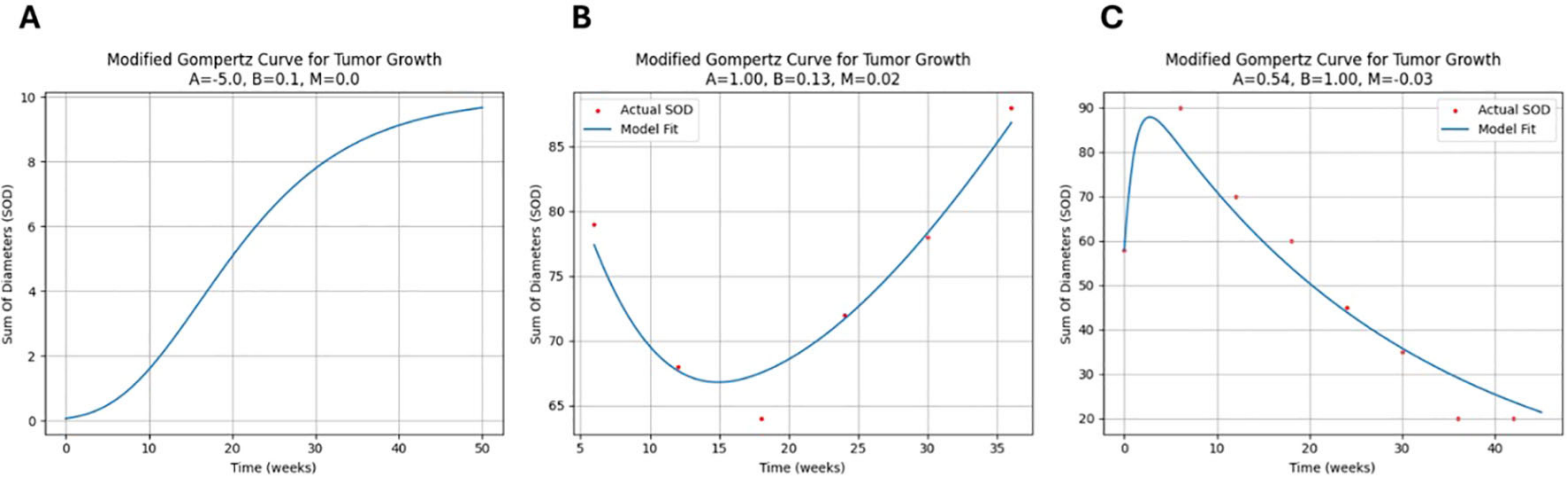
Novel Gompertz model fit samples: **(A)** Typical logistic pattern: The growth rate is initially slow, then increases exponentially before decreasing and approaching zero. **(B)** Tumor growth in a patient with a non-durable response to treatment: The SOD initially decreases, but then increases again. **(C)** Pseudo-progression pattern: The SOD of the tumor initially increases, but then decreases after a period of time.

In this study, a novel version of the Gompertz model was used to describe tumor growth kinetics, tailored to accommodate the observed tumor growth patterns in Immuno-oncology (IO) such as pseudo progression, stagnation phase or hyper progression phase (**Figure 3**).

**Figure 4:**
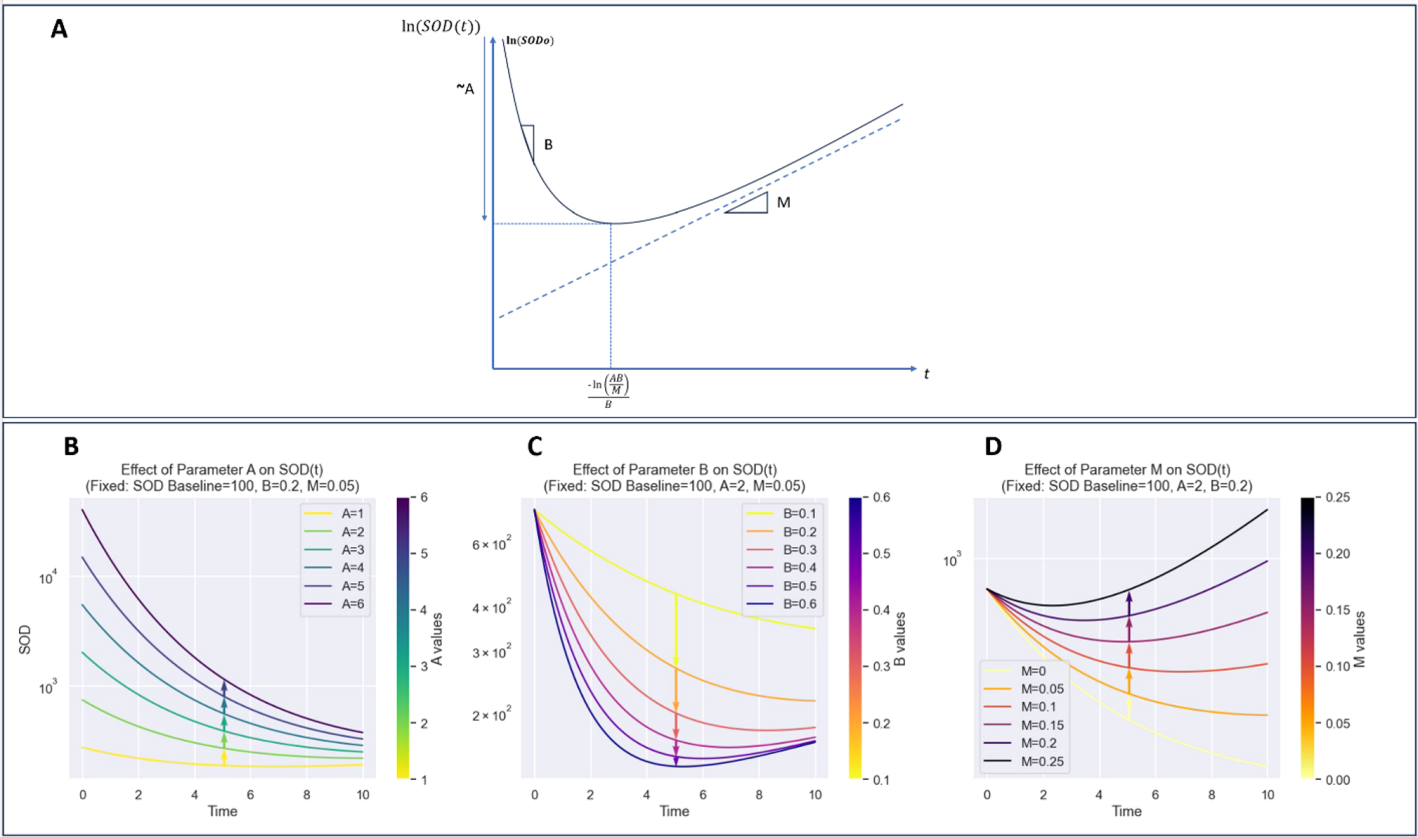
Detailed visualization of model components and their contributions to curve dynamics. **(A)**: Plot of ln(SOD(t)) illustrating the effect of parameters on the overall shape of the function. **(B)**: Impact of parameter A, highlighting its role in defining the curve’s initial slope and growth behavior. **(C)**: Contribution of parameter B, showing its influence on curve inflection points and transitional dynamics. **(D)**: Role of parameter M, emphasizing its effect on curve stabilization and long-term behavior. This breakdown provides insight into how each parameter shapes the model’s behavior and dynamics.

The novel Gompertz model parameters (A, B, M) are designed to capture distinct biological aspects of tumor response. Parameter A represents the depth of response and initial growth rate limitation, often associated with carrying capacity. Parameter B characterizes the speed of response, specifically the decay rate of growth. The novel parameter M captures long-term growth modulation, reflecting immune escape tendency or treatment acquired resistance over time for example. When M > 0, A indicates depth of response, B denotes the speed of response, and M represents resistance to treatment or immune evasion. Conversely, when M<0, A indicates the peak of pseudo-progression, B inversely relates to its duration, and M signals long-term decline (see **Figure 4**).

The novel Gompertz model was used based on its ability to accurately describe the growth patterns observed in the data comparing its predicted values to the observed values using mean absolute error (MAE).


$$SOD(t)=SOD_{o}\times e^{Ae^{-Bt}+Mt}$$

*Where*:


$$SOD_{o}=\frac{SOD_{baseline}}{e^{A}}$$

The growth rate (GR) and decay, when negative, was expressed in mm/months and obtained by taking the derivative of the novel Gompertz model with respect to time. The kinetic parameters A, B, and M were estimated by fitting the model to the early phase time points (up to two follow-up time points) using nonlinear regression.

The adapted model incorporates also an additional linear term with parameter M, allowing for the representation of a constant growth component alongside the typical Gompertzian decay. This modification aims to capture factors influencing decays, where tumor growth exhibits immune escape tendency or treatment effects over time for example. Finally, in our model formulas, the baseline SOD is normalized by dividing it by the exponential of the carrying capacity parameter, *exp(A)*, to account for the system's carrying capacity.

#### 2.2.3 Statistical analysis

The model was fitted to early phase to up to 2 follow-up timepoints, GR at the last follow-up timepoint used for fitting and model parameters A, B, M were collected for each patient and used for descriptive, time to event and for late pattern discrimination analysis. Late vs early pattern discrimination comparison was used on patients with at least seven follow-up timepoints. All statistical analyses were conducted in python 3.11.

##### 2.2.3.1 Descriptive analysis

The above early kinetic parameters of the TGM i.e., A, B, and M, as well as the growth rate (GR), were analyzed descriptively on all patients and according to RECIST 1.1 early phase response patterns defined in **Table 2**.

##### 2.2.3.2 Time to event predictive analysis

The median value of each covariate was used to determine high and low subgroups. Weibull survival analysis was performed to analyze time-to-event data and parameters were estimated via maximum likelihood estimation. The impact of covariates on survival times was also analyzed using the Weibull AFT model. The time-to-event endpoints analyzed were time to response (TTR), and progression-free survival (PFS). The Weibull survival model was selected due to its ability to fit a variety of survival distributions, including those with hazard rates that increase, decrease, or remain constant over time (24, 25) Proportional hazard assumption was tested using Schoenfeld residuals. All survival analysis were performed using lifelines Python package v0.28.

##### 2.2.3.3 Overall response and late response predictive analysis

To investigate the predictive value of early tumor growth parameters for later response patterns, we performed a series of box plots and t-tests comparing the early kinetic parameters between different late phase response groups defined in **Table 2**.

## 3 Results

The novel Gompertz model showed a good fit to the early phase tumor growth data during the first two follow-up time points of treatment, with a mean absolute error (MAE) of 0.767 mm and a standard deviation of 2.356 mm. The growth rate and model parameters were derived from this fit, and were used in the subsequent analyses.

### 3.1 Descriptive analysis GR and early kinetic parameters

**Figure 5:**
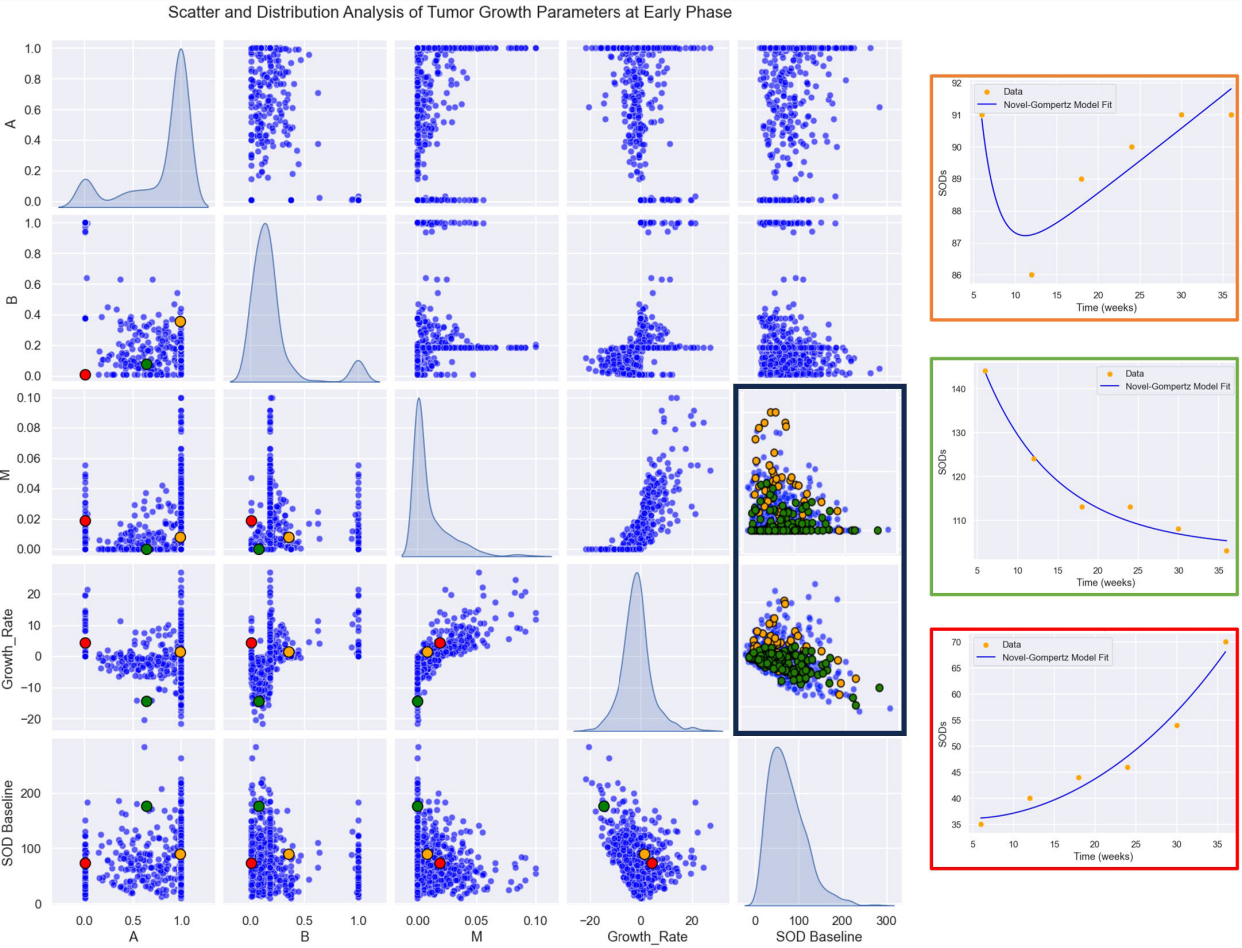
Parameter Relationships and Distributions: A plot matrix showcasing the relationships between parameters (A, B, M, GR). The diagonal elements represent histograms for the distributions of each parameter, providing insights into their variability and range, while the off-diagonal plots illustrate pairwise correlations and interactions between the parameters. Highlighted patients in left: Patient 1 (in orange), early responder with a non-durable response. Patient 2 (in green), durable responder. Patient 3 (in red), progress. Highlighted responses in right: In green durable response. In orange, non-durable response.

We first conducted a descriptive analysis of tumor growth kinetics in the study population as displayed in **Figure 5**. Within the population, the distribution of the GR is normal with an estimated mean GR [ Mean: -0.75, SD: 8.44. mm/week]. A, B and M parameters don’t have a normal distribution, this difference is encouraging to uncover discriminative significance for those kinetic parameters.

**Figure 6:**
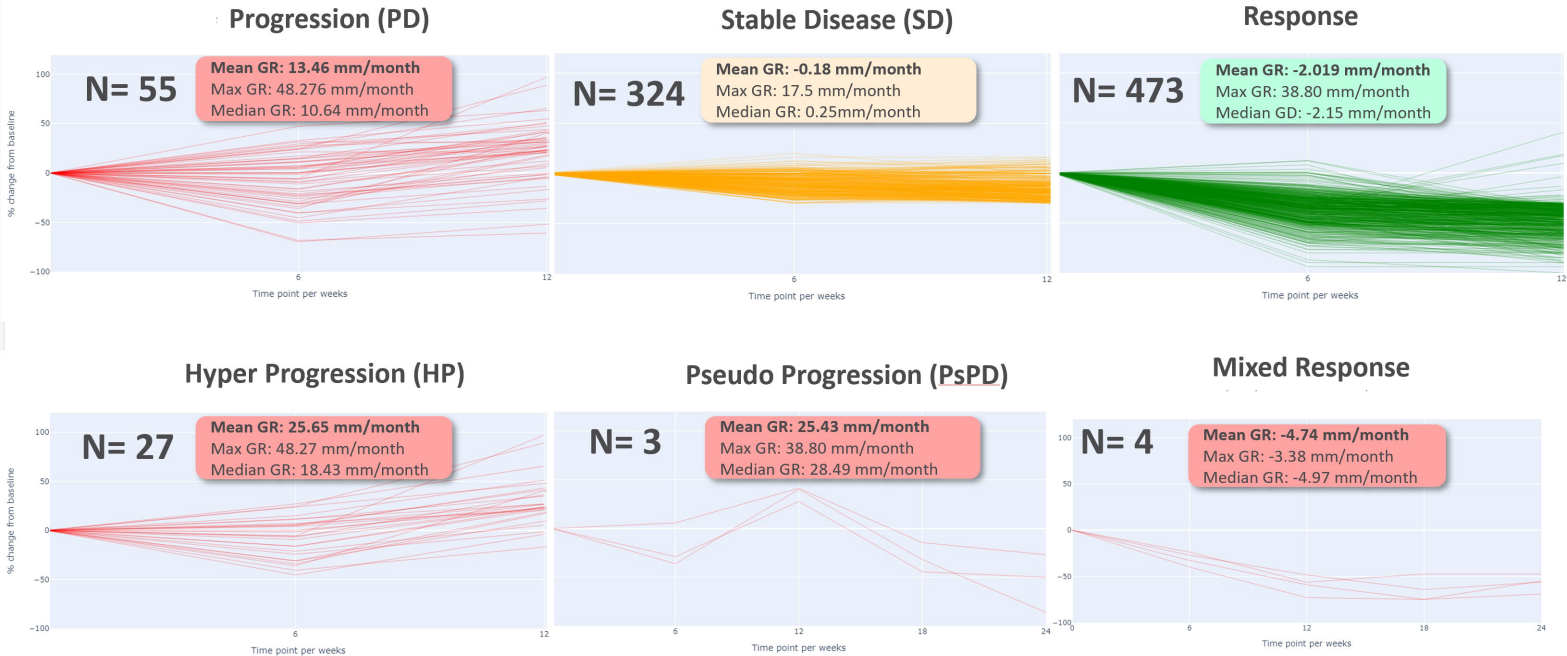
Early phase GR analysis per each type of response pattern.

Then, we computed growth rate (GR) and model parameters for each response profiles at early phase (two follow-up time points) defined in **Table 2**. The results of the descriptive analysis of tumor growth kinetics revealed distinct patterns in growth rate (GR) and model parameters A, B, and M across different response profiles. Progressor patients had the highest GR, with hyper progressors exhibiting the highest mean GR of 25.65 ± 9.38 mm/month, followed by early progressors (13.46 ± 10.60 mm/month) and pseudo-progressors (25.43 ± 12.36 mm/month). In contrast, responder patients had the lowest GR, with early responders exhibiting the lowest mean GR of -2.02 ± 4.31 mm/week, followed by stable disease (-0.18 ± 3.42 mm/month). Interestingly, mixed responders had a notably low GR, like early responders (-4.74 ± 0.87 mm/month) (see **Table 3**, **Figure 6**).

**Table 3:** Mean ± Standard Deviation of Growth Rate (GR) and Model Parameters (A, B, M) for Different Patient Response Types GR (mm/month) A (unitless) B (unitless) M (unitless).

	GR	A	B	M
Paradoxal response	-4.61 ± 0.91 mm/month	1.00 ± 0.00	0.11 ± 0.02	0.00 ± 0.00
Early responder	-1.93 ± 3.60 mm/month	0.89 ± 0.27	0.15 ± 0.14	0.01 ± 0.02
Hyper progressor	9.97 ± 3.03 mm/month	0.16 ± 0.36	0.64 ± 0.45	0.04 ± 0.02
Durable response	-0.09 ± 3.97 mm/month	0.71 ± 0.37	0.26 ± 0.29	0.01 ± 0.02
Pseudo progressor	-2.25 ± 3.0 mm/month	0.51 ± 0.49	0.01 ± 0.00	0.00 ± 0.00
Early progressor	5.15 ± 5.58 mm/month	0.22 ± 0.39	0.64 ± 0.45	0.03 ± 0.02

The comparison of response patterns reveals significant differences in growth rate and parameter values among most of the different response patterns (**Supplementary Table 1**). The parameter A was inversely related to GR, with progressor patients exhibiting the lowest mean A values and responder patients exhibiting the highest. Specifically, early responders had the highest mean A value of 2.99 ± 1.11, while hyper progressors had the lowest mean A value of 0.84 ± 0.97. The parameter B was highest in the hyper progressor pattern (2.25 ± 1.46) and lowest in the mixed response pattern (0.62 ± 0.53). The parameter M was highest in the pseudo progressor pattern (0.33 ± 0.10) and lowest in the mixed response pattern (0.02 ± 0.05) (**Supplementary Table 1**).

### 3.2 Time to event prediction

Our study employed a Weibull model to analyze time-to-event data, investigating the influence of growth rate (GR), parameters A, B, and M on tumor progression and response.

Patients were stratified into two groups based on the median value as the cutoff point of each covariate, distinguishing between high and low levels for subsequent comparative assessment.

**Figure 7:**
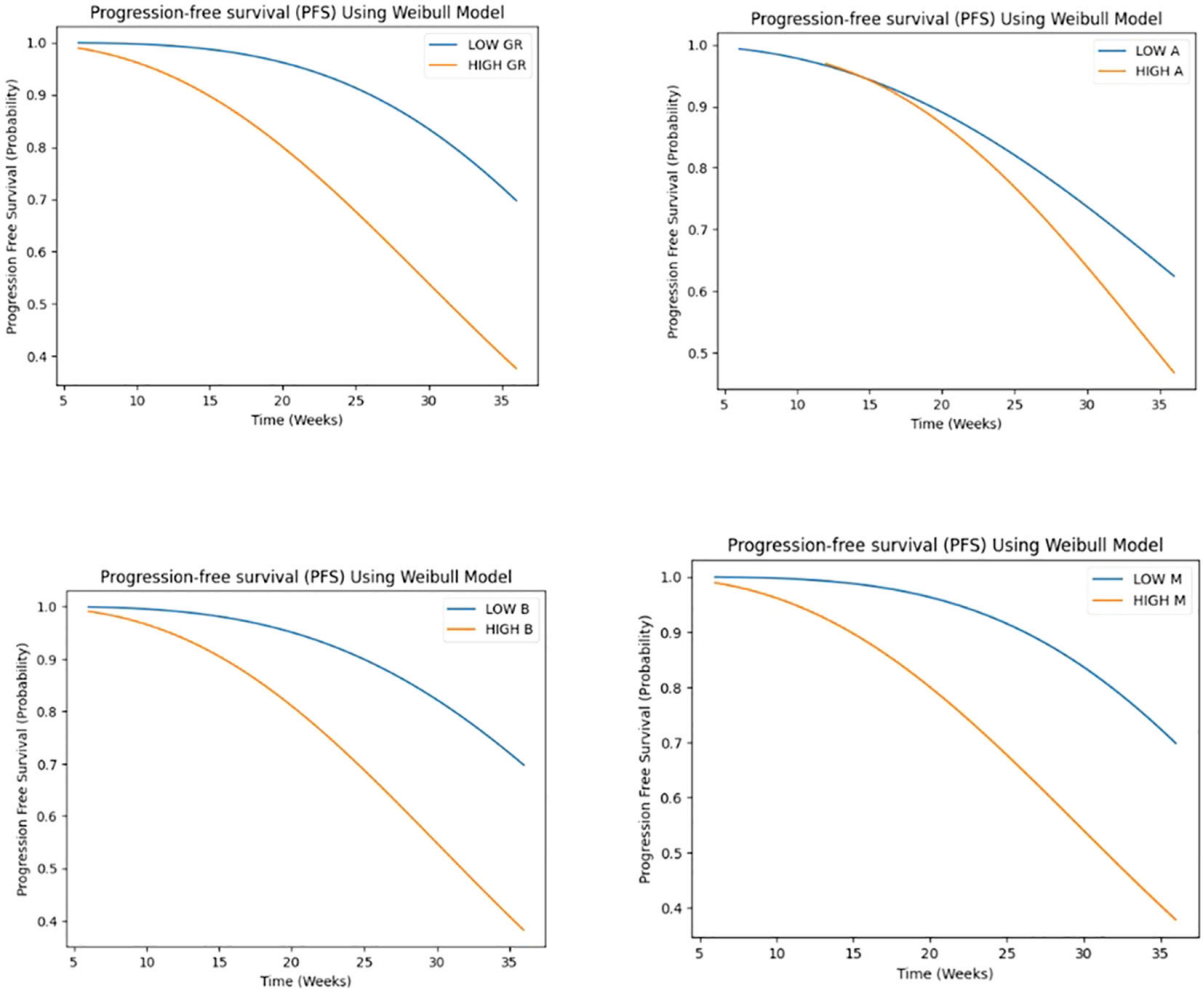
Weibull regression analysis for progression-free survival (PFS) for GR and A, B, M parameters.

The results of the Weibull regression analysis for progression-free survival (PFS) in **Figure 7**, showed that the parameter M had the strongest association with PFS, with a coefficient of -14.11 and a hazard ratio of 7.47 x 10^-7. This indicates that a decrease in the value of M is associated with a significant decrease in the risk of progression. The parameter GR (growth rate) was also significantly associated with PFS, with a coefficient of -0.11 and a hazard ratio of 0.897. This suggests that a lower growth rate is associated with a longer PFS. The parameter B was negatively associated with PFS, with a coefficient of -0.26 and a hazard ratio of 0.773, although the p-value was borderline significant (p< 0.05). The parameter A was not significantly associated with PFS (p>0.05). Overall, these results suggest that the early phase tumor growth kinetics parameters M and GR may be useful predictors of PFS in patients with non-small cell lung cancer.

**Figure 8:**
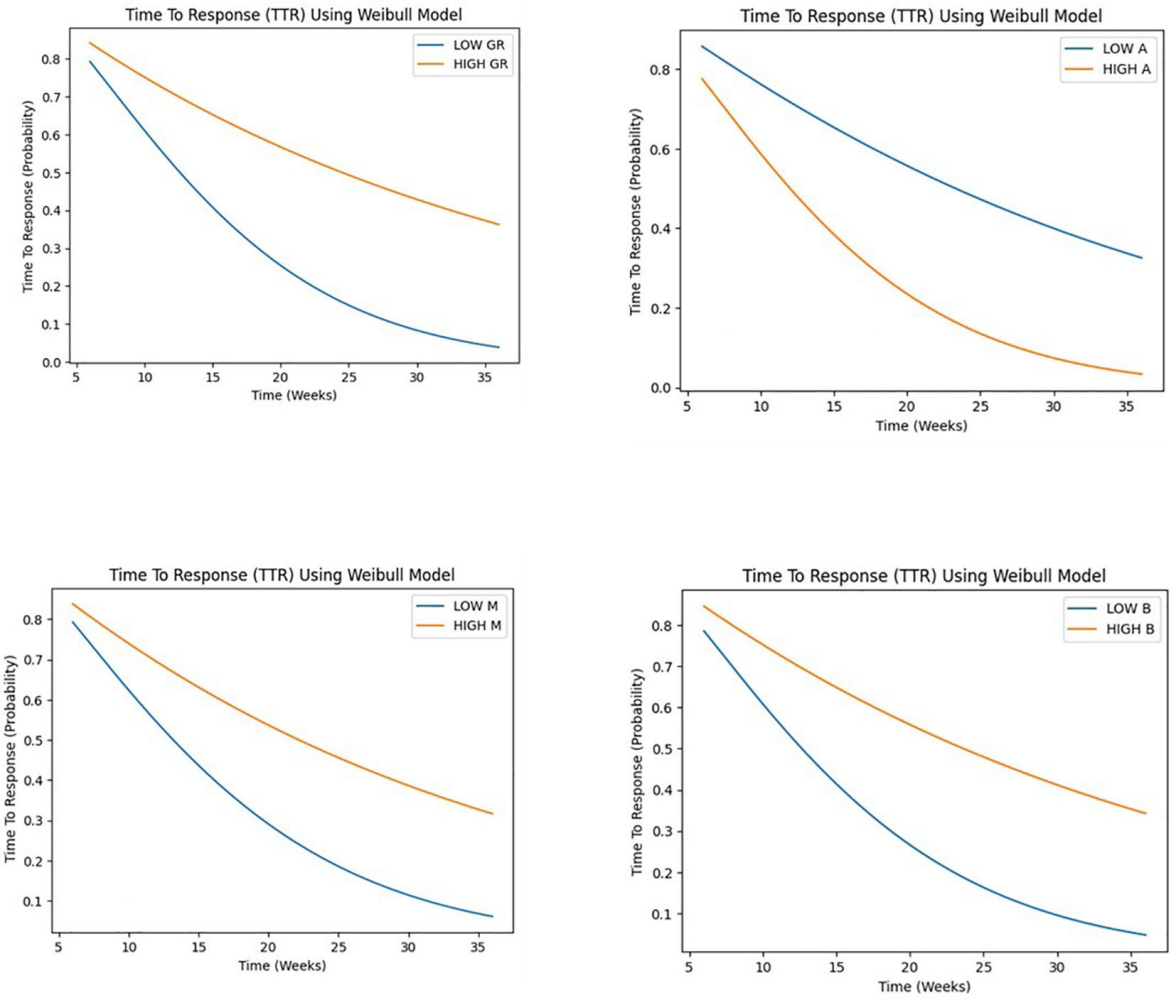
Weibull regression analysis for time to response (TTR) for GR and A, B, M parameters.

GR demonstrated a highly significant association (p < 0.001) with the time-to-event outcome, suggesting that higher GR values are associated with a shorter time to response, with a hazard ratio of 0.575 (95% CI: 0.489 - 0.677), indicating a 42.5% reduction in the hazard rate per unit increase. A marginally significant association (p < 0.05) was observed for A, with one-unit increase in A being associated with a 30.4% reduction in the hazard rate, assuming a Weibull distribution. suggesting that higher A values are also associated with a shorter time to response. Conversely, no significant associations were found for B and M. Despite observing a trend toward shorter time to response with higher B values and longer time to response with higher M values (see **Figure 8**).

### 3.3 Overall response and late response prediction

**Figure 9:**
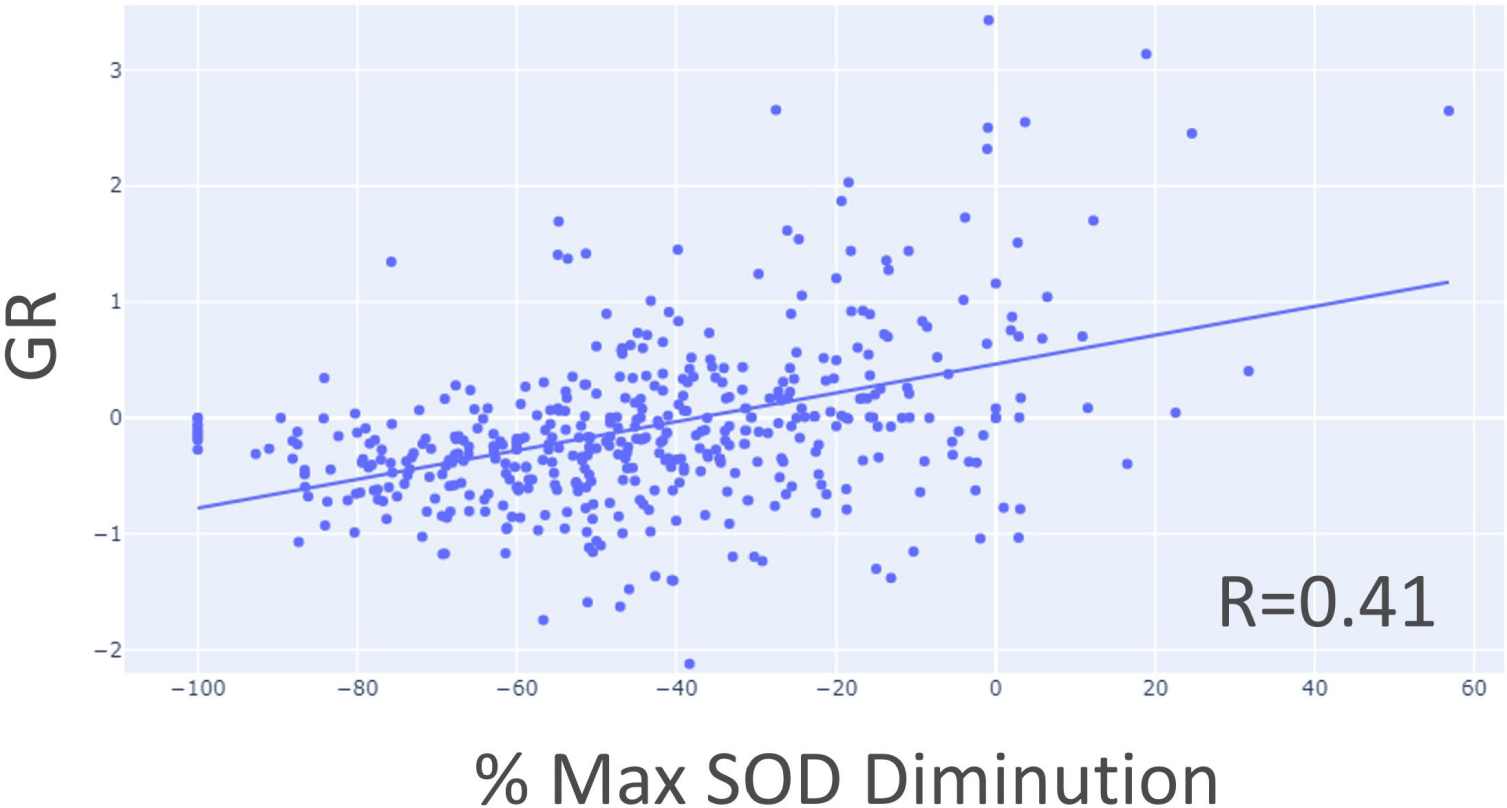
Correlation between growth rate (GR) and maximum SOD reduction percentage using target lesion data only.

**Figure 10:**
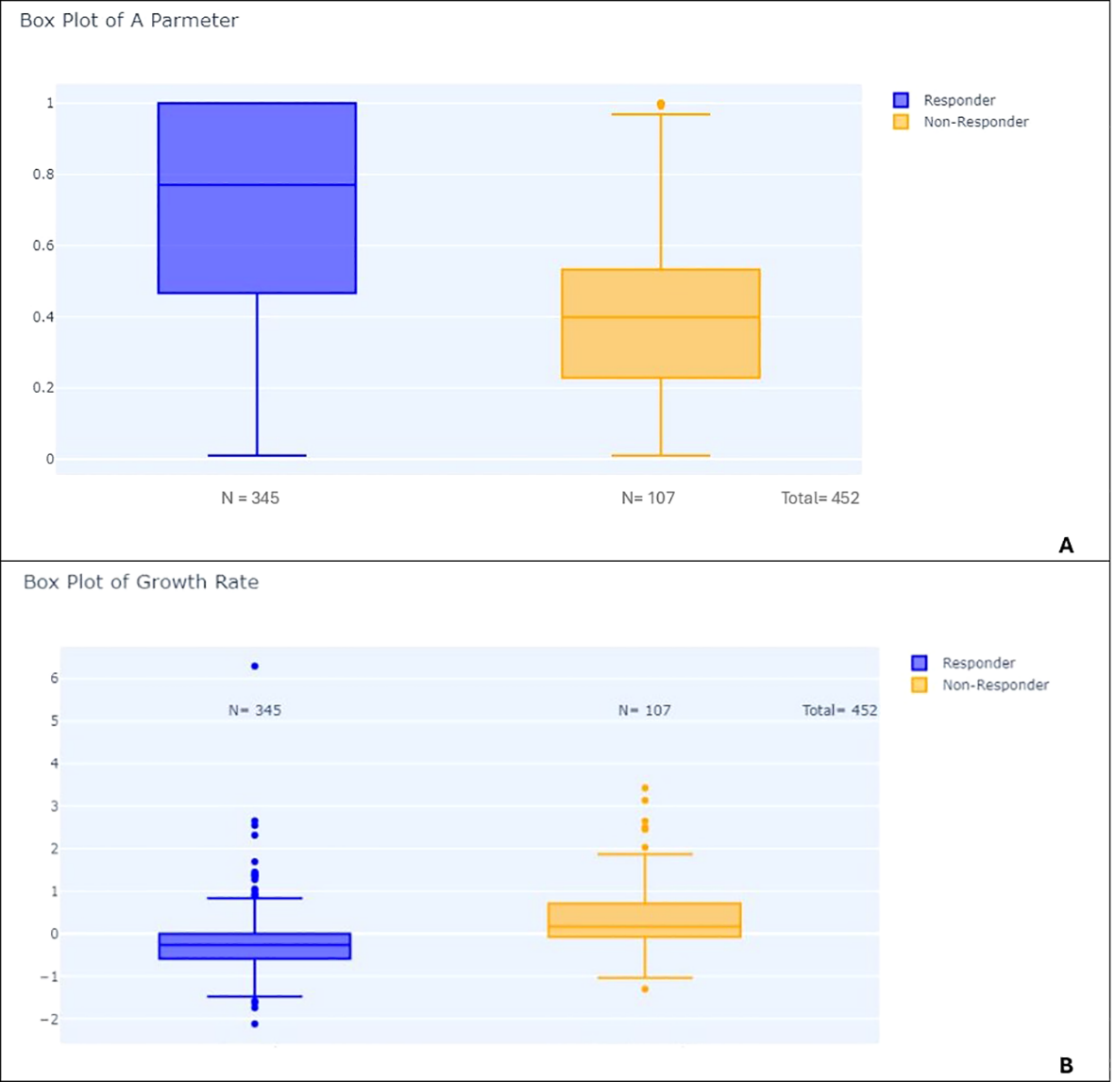
Box plots comparing responders and non-responders: **(A)** Distribution of parameter A in responders vs. non-responders. **(B)** Distribution of growth rate (GR) in responders vs. non-responders.

**Figure 11:**
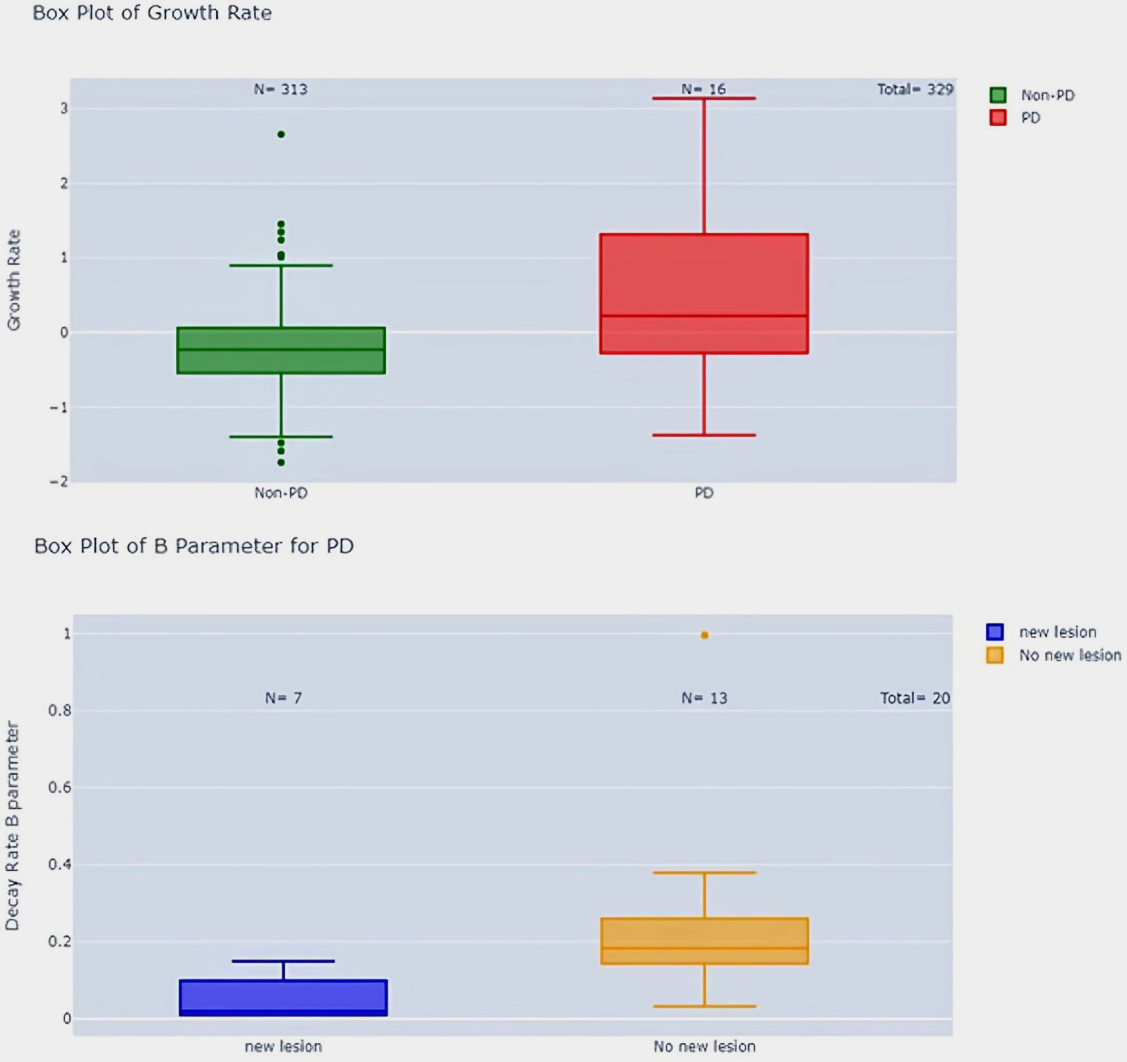
Growth rate comparison in PD vs. Non-PD patients and parameter B in PD patients with and without new lesions.

**Figure 12:**
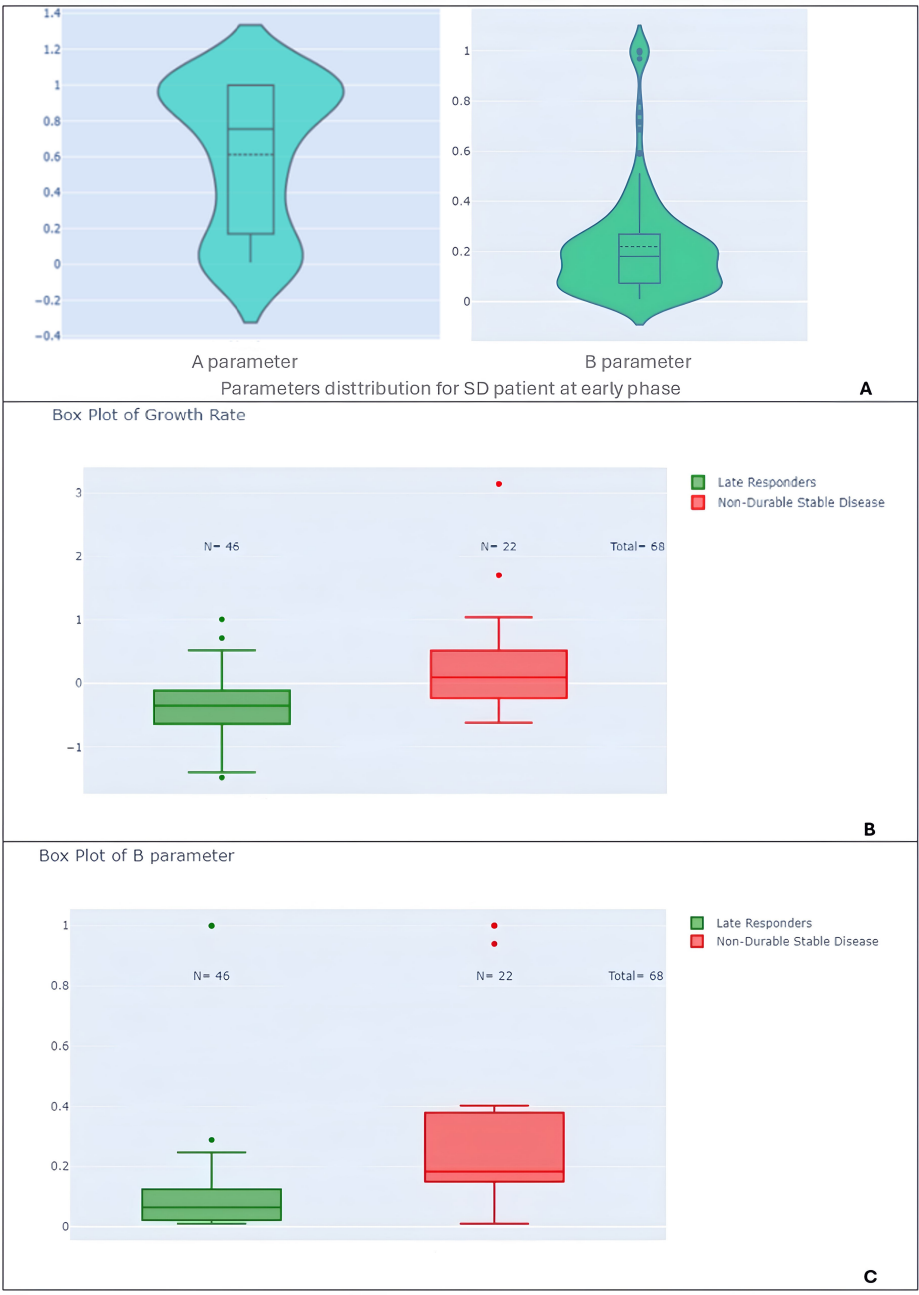
Violin and box plots of model parameters by response subtypes. **(A)** Violin plots of parameter A and B for the entire study population. **(B)** Box plots of growth rate (GR) comparing late responders to non-durable stable disease patients, highlighting distinctions in growth behavior. **(C)** Box plots of parameter B comparing late responders to non-durable stable disease patients.

To investigate the predictive value of early tumor growth parameters for later response patterns, we performed box plots and t-tests comparing the parameters A, B, and M, as well as the growth rate (GR), between different response groups. Only the most significant differences are discussed in **Figures 9**–**12**.

First, it’s interesting to note that we demonstrated a linear relation between early phase GR and best tumor burden shrinkage. The most “active” tumor seems to be most responsive to treatment (**Figure 9**).

Second, we showed significant differences between RECIST overall response groups (PD, SD, CR/PR). Responders had significantly lower A parameters than non-responders (p-value < 0.001) and higher growth rates (p-value < 0.001) (see **Figure 10**). Patients with progressive disease (PD) had higher growth rates compared to patients with non-PD (p-value < 0.001) (**Figure 11**).

Third, we can also distinguish two groups of stable patients within Parameters A and B distribution (see **Figure 12A**). The bimodal distribution of stable patient is confirmed when comparing late patterns for stable disease, patients with non-durable stable disease had higher B parameters (p-value < 0.001) and growth rates (p-value < 0.001) compared to late responders (**Figures 12B, C**).

Additionally, PD patients without new lesions had higher B parameters compared to PD patients with new lesions (p-value < 0.05) (**Figure 11**). We could not find any correlation in regard to pseudo-progression (p-value > 0.05) most probably linked to the lack of this event in our population of study.

## 4 Discussion

Immunotherapy has emerged as a promising treatment option for various types of cancer, offering the potential for durable responses and improved survival outcomes. However, the response to immunotherapy can be highly complex, and there is currently a lack of validated models for predicting treatment outcomes, particularly in comparison to traditional chemotherapy. This lack of prediction models highlights the need for complimentary and reliable approaches that can guide treatment decisions and predict patient outcomes.

In our study, we employed a novel version of the Gompertz model to describe tumor growth kinetics in the context of immunotherapy. This decision was motivated by the model's ability to fit the observed growth patterns in immuno-oncology, including sustained growth tendencies and external influences on tumor growth (26, 27). Most of previous studies correlate growth rate to overall survival (OS) (10, 27–31). Our study investigated the relationship between various covariates (GR and model parameters) with progression-free survival (PFS), time to response (TTR).

The time-to-event analysis revealed that low GR was associated with longer PFS, while high GR was associated with shorter TTR. These findings are consistent with previous studies that have reported an inverse relationship between tumor growth rate and survival outcomes in cancer patients (10, 28). Interestingly, we found that the parameters (B and M) were also predictive of PFS and A to TTR, highlighting the potential of these kinetic parameters as prognostic markers.

Furthermore, results showed notable distinctions in tumor growth dynamics across diverse patient subgroups within the first two follow-up time points after treatment initiation. Specifically, responders exhibited substantially lower A parameters than non-responders (p-value < 0.001), suggesting the potential predictive value of this parameter in identifying treatment responders. Conversely, patients with progressive disease (PD) displayed higher growth rates compared to those without PD (p-value < 0.001), indicating a more aggressive disease trajectory in this subgroup.

The RECIST definition of early stable disease encompasses a heterogeneous population, with some patients experiencing durable responses while others have non-durable responses. When we looked at the GR distribution of the patients in the study two distinct groups stand out.

Being able to discriminate two kinds of stable patient early enough is maybe the most practical finding of this study. In the context of stable disease, patients with non-durable stable disease displayed elevated B parameters (p-value < 0.001) and growth rates (p-value < 0.001) compared to late responders, suggesting an association between these parameters and treatment durability.

Our study has several limitations that should be acknowledged. Firstly, we did not have access to overall survival data, which is an important endpoint in oncology clinical trials. Secondly, the small number of patients exhibiting pseudo-progression and hyper-progression patterns limits the statistical power of our analysis for these subgroups. Also, this study's use of three time points may introduce bias by excluding patients who show limited therapy response. Additionally, it is important to note that while our study aimed to evaluate the predictive value of early tumor growth kinetics for immunotherapy response, it is not possible to fully evaluate the cause of the observed effects while being blinded from the treated arm.

## 5 Conclusion

In conclusion, our study demonstrates the utility of the novel Gompertz model in describing tumor growth kinetics in the context of immunotherapy and identifies important covariates associated with PFS, TTR, and DOR in NSCLC patients. Our findings suggest that early tumor growth parameters may have predictive value for later response patterns and long-term outcomes.

Further prospective studies are needed to validate our findings on different tumor types to more unravel the complex interplay between tumor kinetics and immunotherapy response.

## Data Availability

The raw data supporting the conclusions of this article will be made available by the authors, without undue reservation.
