# Supplementary material for: Assessing immunotherapy response: going beyond RECIST by integrating early tumor growth kinetics
Source: Front Immunol. 2024 Dec 20;15:1470555. doi: 10.3389/fimmu.2024.1470555 (PMC11695367; doi:10.3389/fimmu.2024.1470555)
Supplement: Supplementary file 1 [file Table1.docx]

Table1 P-values for Pairwise Comparisons of Tumor Growth Parameters Across Response Patterns

| **Response Patterns** | | **GR** | **A** | **B** | **M** |
| --- | --- | --- | --- | --- | --- |
| Paradoxical response | Early  responder | **0.00553819** | 0.07324982 | 0.56114054 | 0.37189472 |
| Paradoxical response | Hyper  progressor | **0.00104979** | **6.4689E-07** | **0.02449639** | **6.911E-05** |
| Paradoxical response | Stable  disease | **0.0155381** | **1.3445E-05** | **0.09977096** | **0.0872984** |
| Paradoxical response | Pseudo-progressor | **0.03262806** | **7.295E-07** | **0.00010237** | **0.0021053** |
| Paradoxical response | Early  progressor | 0.08447761 | **4.3997E-09** | **0.01935969** | **0.00038968** |
| Early  responder | Hyper  progressor | **2.9305E-91** | **4.752E-21** | **5.5494E-35** | **3.8139E-59** |
| Early  responder | Stable  disease | **2.3626E-10** | **4.5675E-43** | **5.655E-29** | **0.17614036** |
| Early  responder | Pseudo-progressor | **3.4303E-24** | **2.042E-05** | **5.5813E-20** | **2.1394E-21** |
| Early  responder | Early  progressor | **1.8613E-68** | **2.8434E-35** | **2.7851E-43** | **7.1169E-65** |
| Hyper  progressor | Stable  disease | **2.6121E-83** | **7.8099E-08** | **1.1165E-07** | **1.2105E-77** |
| Hyper  progressor | Pseudo-progressor | 0.54186955 | 0.29977012 | 0.1454814 | 0.10605916 |
| Hyper  progressor | Early  progressor | **0.00114871** | 0.71252091 | 0.46730201 | 0.08759051 |
| Stable  disease | Pseudo-progressor | **1.9552E-28** | **0.00277608** | **9.5437E-06** | **1.1811E-40** |
| Stable  disease | Early  progressor | **1.6594E-53** | **8.3321E-12** | **1.9706E-08** | **2.2766E-73** |
| Pseudo-progressor | Early  progressor | 0.06873341 | 0.18530723 | **0.04407863** | **0.02129297** |
